# Breaking the Growth–Defense Trade-Off: bHLH Transcription Factors as Integrators of Development, Metabolism, and Yield in *Artemisia annua*

**DOI:** 10.3390/genes17080883

**Published:** 2026-07-29

**Authors:** Mingyuan Yuan, Fei Zhou

**Affiliations:** State Key Laboratory of Pharmaceutical Biotechnology, School of Life Sciences, Nanjing University, Nanjing 210023, China; ymyitj@smail.nju.edu.cn

**Keywords:** artemisinin, bHLH transcription factors, glandular secretory trichomes, hormone crosstalk, metabolic engineering, whole-plant yield

## Abstract

Artemisinin, a sesquiterpene lactone produced by *Artemisia annua*, is synthesized and stored predominantly in glandular secretory trichomes (GSTs). Despite substantial advances in elucidating its biosynthetic pathway and transcriptional regulation, a key challenge remains: how developmental processes, hormone signaling, and metabolic pathways are coordinately integrated to overcome the trade-off between trichome density, metabolic flux, and vegetative biomass, which ultimately limits whole-plant yield. Recent studies have identified basic helix–loop–helix (bHLH) transcription factors (TFs) as central integrative hubs in this network. AabHLH113 functions as a convergence node linking jasmonic acid (JA) and abscisic acid (ABA) signaling to activate core biosynthetic genes. AaMYC3 bridges development and metabolism by promoting GST initiation via AaHD1 while enhancing pathway flux and cooperating with other bHLH factors to amplify amorpha-4,11-diene synthase (*ADS*) and artemisinic aldehyde delta-11(13) reductase (*DBR2*) expression. Extending this regulation to the whole-plant level, AaSPATULA coordinates GST formation with photosynthetic capacity, carbon assimilation, and biomass accumulation. Here, we propose a unified framework in which bHLH TFs integrate hormone signaling, trichome development, metabolic flux, and carbon allocation into a coherent regulatory system. We further discuss implications for next-generation metabolic engineering and highlight future directions, including multi-omics-guided, multi-target editing of regulatory hubs. This developmental–metabolic integration framework provides a conceptual basis for optimizing artemisinin production and improving high-value metabolite biosynthesis in other trichome-bearing plants.

## 1. Introduction

As sessile organisms, land plants are constantly exposed to diverse biotic and abiotic stresses. To cope with these challenges, plants have evolved a variety of responses; notably, they produce bioactive secondary metabolites that act as chemical defenses against herbivores and pathogens [1,2,3]. Phytohormones, particularly jasmonic acid (JA) and abscisic acid (ABA), play central roles in regulating the biosynthesis of these metabolites [4,5,6,7,8]. For example, JA promotes paclitaxel production in *Taxus*, alkaloid accumulation in *Dendrobium officinale*, and volatile terpenoid biosynthesis in *Lavandula angustifolia*, whereas ABA enhances flavonoid biosynthesis in tomato (*Solanum lycopersicum*), epidermal wax accumulation in apple (*Malus domestica*), and terpenoid production in *Cannabis sativa* [9,10,11,12,13,14].

Rather than acting through isolated linear pathways, JA and ABA operate within interconnected regulatory networks mediated by transcription factors (TFs), forming synergistic or antagonistic modules that fine-tune development, stress responses, and secondary metabolism [15,16,17]. Glandular trichomes are key components of this system, serving both protective roles and as primary sites for secondary metabolite biosynthesis [18,19,20,21]. While non-glandular trichomes (NGTs) mainly provide mechanical defense, glandular secretory trichomes (GSTs) function as specialized factories for producing and storing terpenoids, alkaloids, and flavonoids. Representative examples include terpene-based defense in tomato GSTs, gossypol accumulation in cotton (*Gossypium* spp.) pigment glands, alkane metabolism in tobacco (*Nicotiana tabacum*), and the storage of diverse metabolites in medicinal plants such as *Tussilago farfara*, *Withania somnifera*, and *Artemisia annua* [22,23,24,25,26,27]. A central unresolved challenge is the trade-off between trichome density and vegetative biomass, which limits the whole-plant yield of targeted metabolites despite enhanced biosynthetic pathway activity.

The molecular mechanisms of trichome development and secondary metabolism are closely interconnected [28]. In *Arabidopsis*, the MYB–bHLH–WD40 (MBW) complex activates the HD-ZIP IV gene *GL2* to promote trichome formation, a regulatory framework largely conserved for NGTs [29,30]. In cotton, tomato and *A. annua*, HD-ZIP genes remain central to GST development [31,32,33]. However, most studies have examined trichome development or metabolic pathways separately, leaving their coordination poorly understood. In *A. annua*, GSTs are predominantly distributed on young leaves, and artemisinin content strongly correlates with leaf biomass and developmental stage [34,35,36,37,38,39], suggesting that leaf growth and trichome formation jointly determine production capacity. Similar links between development and specialized metabolism have been reported in several other species [40,41,42,43,44], yet the molecular basis of this coordination in *A. annua* remains largely unclear.

In *A. annua*, artemisinin is predominantly synthesized and stored in GSTs [27,36]. Although increasing GST density can elevate artemisinin content, it often reduces vegetative biomass, creating a classical “high trichome–low biomass” trade-off that constrains whole-plant yield [33,45,46]. While numerous TFs from bHLH, ERF, MYB, and WRKY families regulate either GST initiation or artemisinin biosynthesis in response to hormonal and environmental cues, the mechanisms coordinating trichome development, metabolic flux, and plant growth remain poorly understood. This regulatory separation and resource allocation conflict represent a major challenge in the field.

It should be noted that this is a narrative rather than a systematic review. Relevant studies were primarily retrieved from the Web of Science, PubMed, and Google Scholar databases using combinations of the keywords “*A. annua*”, “artemisinin”, “bHLH transcription factor”, “glandular trichome”, “jasmonic acid”, and “abscisic acid”. Priority was given to recent peer-reviewed publications, together with landmark articles that established the current understanding of artemisinin biosynthesis and glandular trichome development.

In this review, we summarize recent advances regarding bHLH transcription factors in hormone signaling, glandular trichome development, artemisinin biosynthesis, and plant growth. Based on current evidence, we propose a conceptual framework illustrating how selected bHLH TFs may coordinate these processes. Although individual bHLH TFs regulate distinct developmental or metabolic events, the collective findings suggest that they serve as regulatory nodes linking hormone signaling, glandular trichome development, metabolism, and vegetative growth.

## 2. Distinct Transcriptional Modules Governing Artemisinin Biosynthesis and Glandular Trichome Development in *Artemisia annua*

Artemisinin, a sesquiterpene lactone containing a unique endoperoxide bridge, is predominantly biosynthesized and stored in the glandular secretory trichomes (GSTs) of *A. annua* [27,36]. These specialized 10-celled structures provide a compartmentalized microenvironment that supports efficient terpenoid production while preventing autotoxicity [47].

The biosynthetic pathway of artemisinin has been largely elucidated. Isopentenyl diphosphate (IPP) and dimethylallyl diphosphate (DMAPP), derived predominantly from the mevalonate (MVA) pathway with possible contributions from the 2-C-methyl-D-erythritol-4-phosphate (MEP) pathway, are condensed by farnesyl diphosphate synthase (FPPS) to generate farnesyl diphosphate (FPP), which is subsequently cyclized by amorpha-4,11-diene synthase (ADS) to form amorpha-4,11-diene [48]. Amorpha-4,11-diene is sequentially oxidized by cytochrome P450 monooxygenase CYP71AV1 to artemisinic alcohol and artemisinic aldehyde [49]. Artemisinic aldehyde is then reduced by artemisinic aldehyde Δ11(13) reductase (DBR2) to dihydroartemisinic aldehyde, which is further oxidized by aldehyde dehydrogenase 1 (ALDH1) to dihydroartemisinic acid (DHAA) [50,51]. Finally, DHAA is converted into artemisinin through spontaneous photooxidation and autooxidation. ADS, CYP71AV1, DBR2, and ALDH1 are the key rate-limiting enzymes that control metabolic flux toward artemisinin biosynthesis.

**Figure 1:**
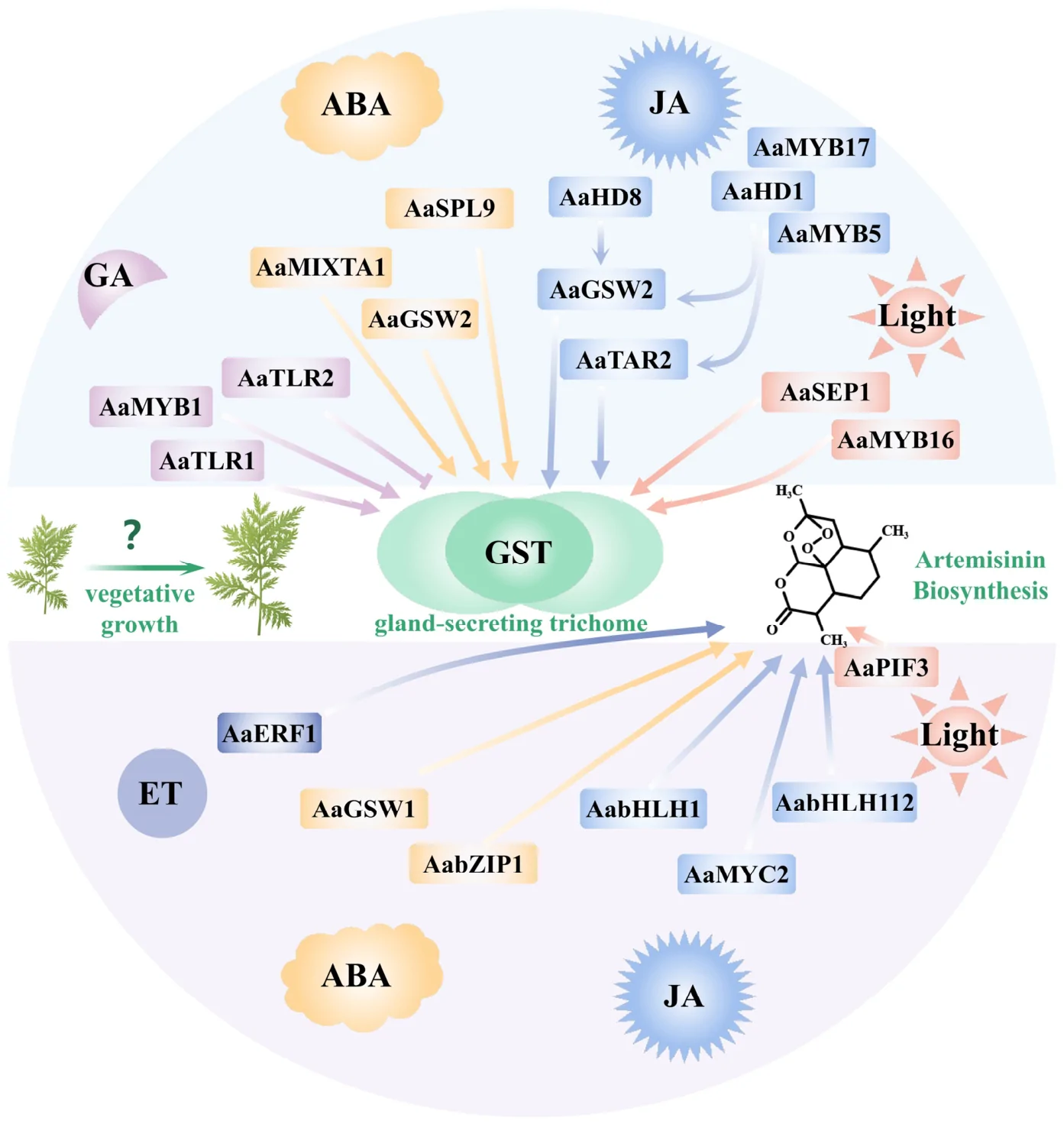
Transcription factors responsive to different signals regulate artemisinin biosynthesis and glandular secretory trichome (GST) development. The upper section illustrates regulatory networks governing the development of artemisinin-producing GST in response to various signals, while the lower section depicts regulatory networks for artemisinin biosynthesis genes under different signals. Regulatory elements associated with vegetative growth in *A. annua* remain unidentified (indicated by question marks); arrows represent the progression of vegetative growth stages. Key signals include gibberellin (GA), abscisic acid (ABA), jasmonic acid (JA), ethylene (ET), and light. Different colors represent distinct hormonal signals or light, with arrows indicating positive regulation and horizontal arrows indicating negative regulation.

Transcription factors (TFs) regulate artemisinin biosynthesis through multi-family regulatory modules. Members from the AP2/ERF (e.g., AaERF1/2, AaORA) and WRKY (e.g., AaWRKY1, AaGSW1) families directly activate the expression of key pathway genes such as *ADS* and *CYP71AV1*, thereby enhancing artemisinin biosynthesis and accumulation [38,52,53,54]. Additional regulators from the TCP, SPL and NAC families further modulate the expression of biosynthetic genes in response to various hormones signals, including gibberellic acid (GA), ABA, JA, ethylene (ET), and light [39,54,55,56,57,58] (Figure 1). Notably, these TFs predominantly act at the metabolic level, with limited involvement in coordinating trichome development.

In contrast, development of GSTs is primarily governed by a distinct transcriptional module centered on MYB and HD-ZIP regulators. MYB factors such as AaMYB1, AaMIXTA1, and AaMYB17 promote trichome initiation and differentiation [43,59,60], while competitive interactions (e.g., AaMYB16 vs. AaMYB5) fine-tune GST formation through modulation of AaHD1 activity [46,61] (Figure 1). HD-ZIP factors, particularly AaHD1 and AaHD8, function as core regulators by activating downstream targets such as *AaGSW2*, thereby establishing the transcriptional framework for GST development [33,61].

Despite substantial progress, most studies have examined trichome development and biosynthetic pathways independently. The coordination between GST initiation, artemisinin biosynthesis, and vegetative growth remains poorly understood. This regulatory fragmentation underscores the need to identify integrative transcriptional hubs, particularly bHLH factors, that bridge development, metabolism, and whole-plant physiology to optimize artemisinin yield.

## 3. Potential Roles of bHLH Transcription Factors in Integrating JA and ABA Signaling

Plant hormones, particularly JA and ABA, play central and interconnected roles in regulating artemisinin biosynthesis and glandular secretory trichome (GST) development in *A. annua*. Exogenous methyl jasmonate (MeJA) treatment markedly enhances artemisinin accumulation by inducing the expression of key biosynthetic genes, including *ADS*, *CYP71AV1*, *DBR2*, and *ALDH1* [62]. Similarly, ABA promotes artemisinin production by upregulating pathway genes such as *AaHMGR*, *AaFPPS*, and *CYP71AV1*, while maintaining GST formation under stress conditions [63]. Rather than acting independently, JA and ABA operate within an interconnected regulatory network, where synergistic or antagonistic interactions fine-tune development, stress responses, and secondary metabolism [15,16].

**Figure 2:**
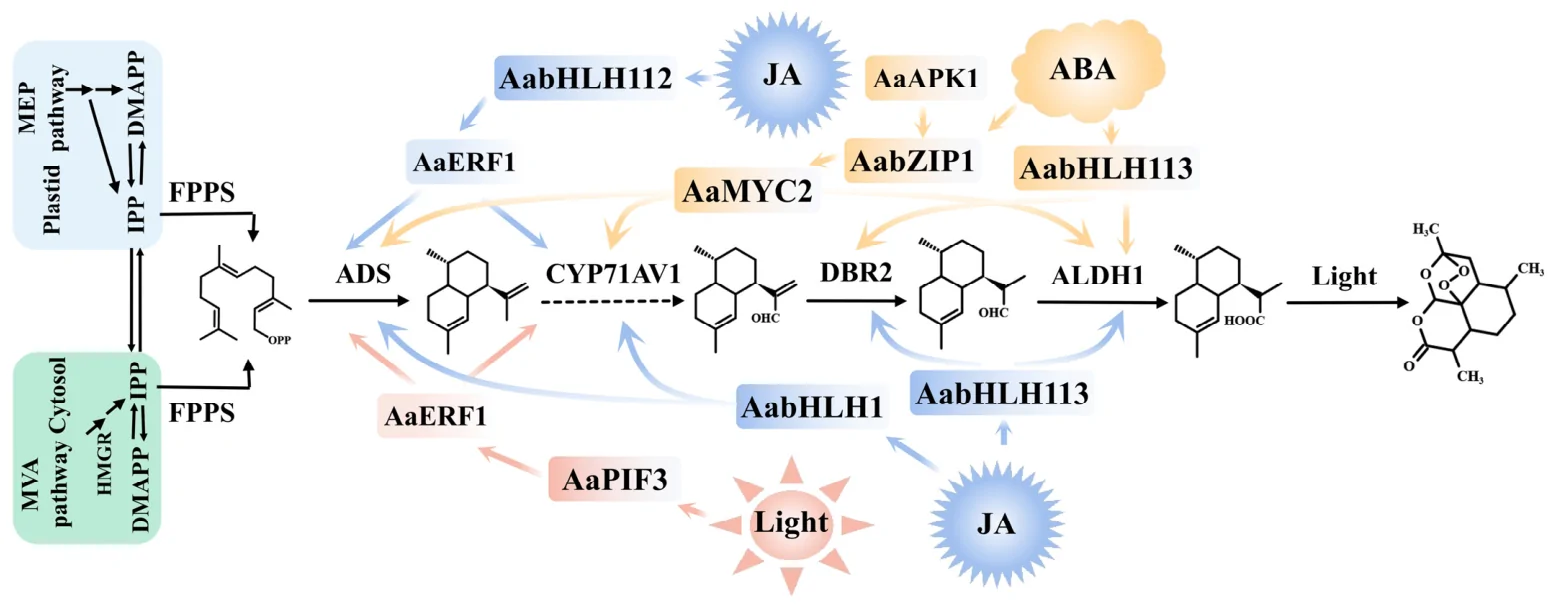
The bHLH transcription factor serves as a core regulatory hub integrating jasmonic acid and abscisic acid signaling pathways. Colored arrows indicate different signaling pathways through which the transcription factor regulates genes involved in artemisinin biosynthesis; black arrows represent catalytic reaction steps of key enzymes in artemisinin biosynthesis; dashed arrows denote multi-step reactions involving the same enzyme. Key signals include abscisic acid (ABA), jasmonic acid (JA), and light.

At the molecular level, this crosstalk is mediated by TFs functioning as signal integration nodes, among which basic helix–loop–helix (bHLH) proteins emerge as central hubs. In JA signaling, core regulators such as AaMYC2 directly activate artemisinin biosynthetic genes via G-box elements, thereby enhancing metabolic flux through the pathway [64,65]. Additional JA-responsive bHLH factors, including AabHLH1 and AabHLH112, further amplify this cascade by directly targeting biosynthetic genes or activating downstream regulators such as *AaERF1* [66,67] (Figure 2). Meanwhile, JA signaling also promotes GST initiation through activation of developmental regulators such as AaHD1, linking hormone perception to structural capacity for metabolite production [33,36].

In contrast, ABA signaling modulates artemisinin biosynthesis through a partially overlapping yet distinct module. The ABA-responsive bZIP TF AabZIP1 directly activates biosynthetic genes and downstream regulators such as *AaMYC2* and *AabHLH113*, while upstream kinase AaAPK1 enhance this pathway via post-translational modification [65,68,69] (Figure 2). In addition, ABA contributes to maintaining GST integrity and cellular homeostasis under abiotic stress, thereby supporting sustained metabolite accumulation [70].

A key emerging concept is that JA and ABA signaling converge not only at individual pathway genes but at shared transcriptional hubs, particularly bHLH factors. For example, AabHLH113 is co-regulated by the JA-responsive AabHLH112 and the ABA-responsive AabZIP1, positioning it as a convergence node that integrates both signals to coordinately regulate downstream biosynthetic genes [69] (Figure 2). More broadly, bHLH TFs integrate hormone signaling through three interconnected mechanisms: (i) direct activation of biosynthetic and developmental genes via E-box/G-box elements, (ii) protein–protein interactions enabling cooperative regulation with other TF families, and (iii) convergence of multiple hormonal inputs onto shared regulatory nodes.

Together, these features define bHLH TFs as multi-layered integrators rather than simple downstream components of hormone signaling, highlighting a shift from linear hormone–gene models toward network-level regulatory coordination.

## 4. Pivotal Roles and Regulatory Constraints of MYC-Type Transcription Factors in Coupling Glandular Trichome Development and Artemisinin Biosynthesis

GSTs are specialized epidermal structures that function as micro-organs for the biosynthesis and sequestration of secondary metabolites, including artemisinin in *A. annua* [21]. In *A. annua*, peltate GSTs provide a compartmentalized environment that supports efficient terpenoid production while preventing cellular toxicity [47]. Notably, GST density is largely determined during early leaf development and positively correlates with artemisinin production capacity [61], highlighting GSTs as the key structural determinants of metabolic output.

**Figure 3:**
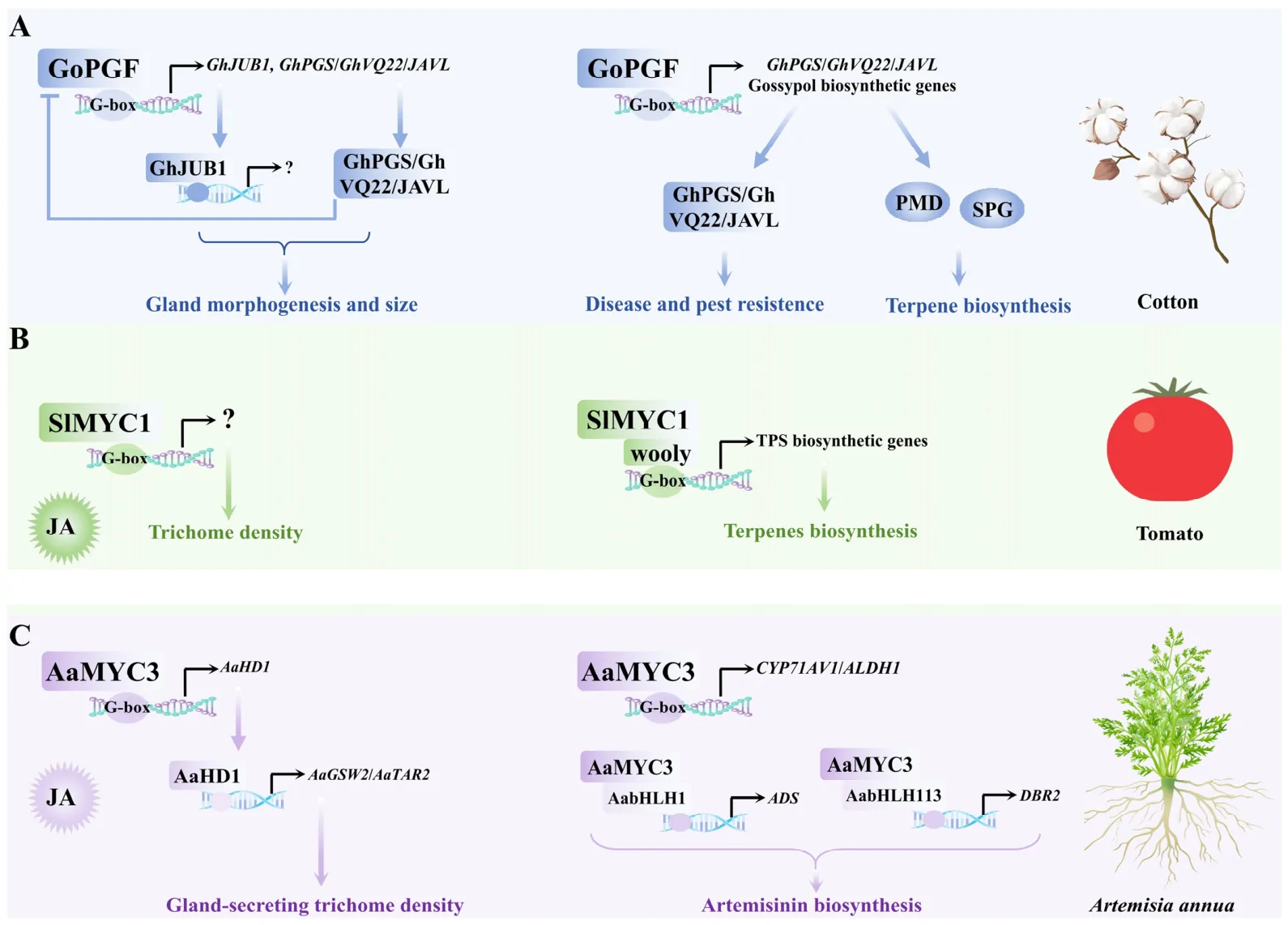
The regulatory mechanism of MYC-type transcription factors in coupling glandular secretory trichome (GST) development with secondary metabolic biosynthesis. In cotton (**A**), tomato (**B**), and *A. annua* (**C**), MYC-type transcription factors simultaneously regulate both GST development and the biosynthesis of terpenoids. The hormone signal depicted in the figure is jasmonic acid (JA). Black arrows indicate gene transcription processes, colored arrows (blue, green, and purple) represent regulatory mechanisms, and black question marks denote downstream unknown gene expressions.

Evidence from multiple species supports a conserved developmental–metabolic coupling. In cotton, the MYC2-like TF GoPGF simultaneously regulates pigment gland formation and gossypol biosynthesis, while its downstream regulatory factor GoPGS exerts negative feedback regulation on the gland development factor GoPGF [71,72] (Figure 3A). Additional components, including GhVQ22 and JAVL, further repress *GoPGF* via JA signaling, linking gland initiation with secondary metabolism [32,71,73] (Figure 3A). Similarly, in tomato, the JA-responsive bHLH factor SlMYC1 coordinates terpene biosynthesis and glandular trichome formation [74,75] (Figure 3B), suggesting that MYC-type regulators can simultaneously control structural and metabolic processes (Figure 3A,B).

In *A. annua*, GST initiation is governed by a hierarchical transcriptional network centered on HD-ZIP regulators such as AaHD1, which activates downstream targets including *AaGSW2* and *AaTAR2* [33,76]. In contrast, artemisinin biosynthesis is primarily controlled by TFs such as AabHLH1 and AabHLH113 that directly activate pathway genes [66,69]. Despite extensive characterization, these modules remain largely uncoupled, representing a major gap in understanding how structural capacity and metabolic flux are coordinated.

AaMYC3, a jasmonate-induced bHLH TF, represents one of the best-characterized examples linking glandular trichome development with artemisinin biosynthesis. It functions as a dual regulatory module: on the developmental side, AaMYC3 directly activates *AaHD1*, promoting GST initiation and increasing trichome density; on the metabolic side, it upregulates key biosynthetic genes such as *CYP71AV1* and *ALDH1*. In addition, AaMYC3 acts as a transcriptional co-activator by interacting with AabHLH1 and AabHLH113, thereby enhancing *ADS* and *DBR2* expression. Through this multi-layered regulation, AaMYC3 coordinates structural capacity (GST abundance) with enzymatic capacity (pathway flux), establishing a direct molecular link between trichome development and specialized metabolism [45] (Figure 3C).

This integrative mode is consistent with MYC-type regulators in other systems, such as SlMYC1 in tomato and GoPGF in cotton [71,74], suggesting a conserved regulatory strategy in glandular tissues. However, AaMYC3 also reveals intrinsic constraints in co-optimizing structure and metabolism. Although its overexpression increases both GST density and artemisinin content, the metabolic gain is relatively moderate compared with pathway-specific regulators [33,64,69,76], likely reflecting limitations in carbon allocation, precursor supply, and pathway feedback regulation.

These observations point to a regulatory ceiling in which increasing structural capacity imposes additional metabolic and energetic demands, leading to competition for carbon resources and constraining further enhancement of pathway flux. Thus, while MYC-type TFs act as integrative nodes linking development and metabolism, their capacity to simultaneously maximize both processes is inherently limited.

Collectively, these findings support a model in which MYC-type bHLH TFs coordinate glandular structure formation and specialized metabolism as part of a conserved regulatory strategy, while also revealing fundamental constraints that must be addressed for effective yield optimization.

## 5. SPATULA-like Transcription Factor Orchestrates Glandular Trichome Development, Artemisinin Biosynthesis, and Whole-Plant Physiological Balance in *A. annua*

Several bHLH TFs have been identified as key regulators of artemisinin biosynthesis in *A. annua*. For example, AabHLH1 directly activates *ADS*, while AaMYC2 binds to G-box motifs in *CYP71AV1* and *DBR2* promoters to enhance artemisinin accumulation [64,66]. Other members, such as AaPIF3 and AabHLH112, also positively regulate this pathway, whereas AabHLH2 and AabHLH3 act as negative regulators, reflecting functional diversification within the bHLH family [77].

Despite these advances, most studies have focused on pathway-level regulation, with limited attention to the coordination between GST development and whole-plant growth. Nevertheless, increasing evidence suggests that bHLH TFs can integrate developmental and metabolic processes. For instance, SlMYC1 in tomato simultaneously regulates glandular trichome formation and terpene biosynthesis, while SPATULA-like proteins in *Arabidopsis* control leaf expansion and biomass accumulation [74,78,79,80,81].

In *A. annua*, GSTs are the primary sites of artemisinin biosynthesis, and their density positively correlates with artemisinin content [36]. However, increasing GST density alone does not proportionally enhance yield, as production is constrained by biomass and carbon availability [34,35]. These observations highlight the importance of whole-plant physiological regulation in determining final artemisinin output.

**Figure 4:**
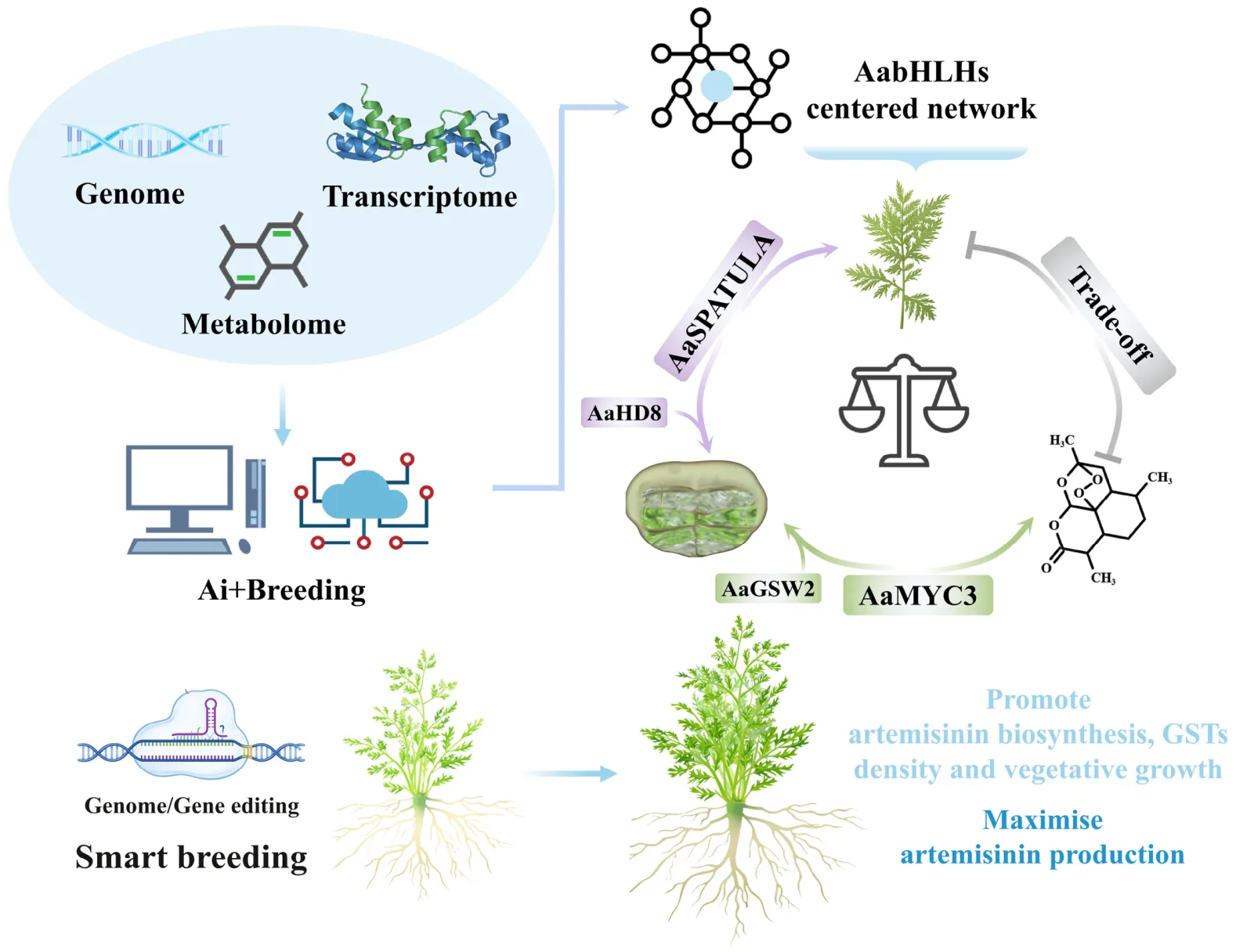
The bHLH transcription factor network represented by AaMYC3 and AaSPATULA reveals the future shift in engineering design toward integrated developmental and metabolic approaches. AaSPATULA coordinates the initiation of glandular secretory trichome (GST) formation and the vegetative growth of *A. annua*, while AaMYC3 simultaneously regulates GST density and artemisinin biosynthesis. However, excessive artemisinin inhibits the vegetative growth of *A. annua*, establishing a trade-off relationship between the autotoxicity of artemisinin and plant development. This highlights the pivotal role of AabHLH transcription factors. By integrating genomic, transcriptomic, and metabolomic data into artificial breeding design, future studies may identify centrally regulated AabHLH transcription factors to breed high-quality *A. annua* varieties with maximum artemisinin yield.

Recent studies identify AaSPATULA as a key integrative regulator linking GST development with vegetative growth. AaSPATULA promotes trichome initiation by interacting with AaHD8 and activating *AaGSW2* [33,61,82]. Notably, it also enhances leaf expansion, photosynthetic capacity, and carbon assimilation, leading to increased biomass and metabolic supply. Transcriptomic and metabolomic analyses further indicate that AaSPATULA upregulates primary carbon metabolism, thereby supporting secondary metabolite biosynthesis [82] (Figure 4).

This dual function distinguishes AaSPATULA from classical pathway regulators. Rather than acting solely on local metabolic flux, AaSPATULA coordinates structural capacity (GST density) with resource availability (carbon and energy supply), resulting in a substantially greater increase in whole-plant artemisinin yield than can be achieved by manipulating GST density alone.

Moreover, artemisinin production is constrained by developmental timing and feedback regulation, including the decline of GST density during leaf maturation and feedback inhibition of key biosynthetic genes [36,61]. Within this context, AaSPATULA provides a mechanism to alleviate the classical “high trichome–low biomass” trade-off by simultaneously enhancing both structural and metabolic capacity [82,83,84,85,86] (Figure 4).

Overall, AaSPATULA represents a key transcriptional hub that integrates glandular trichome development, primary metabolism, and artemisinin biosynthesis. This system-level regulatory model shifts the focus from pathway-centric control to whole-plant resource allocation, offering a promising strategy for improving artemisinin yield through coordinated developmental and metabolic engineering.

## 6. Conclusions and Future Perspectives

bHLH transcription factors (TFs) have emerged as central regulatory hubs in *A. annua*, offering promising targets for metabolic engineering aimed at improving whole-plant artemisinin yield. Traditional strategies based on overexpressing individual enzymes or TFs can increase pathway flux but are often constrained by a trade-off between GST density and vegetative biomass, ultimately limiting yield per plant [45,46]. These limitations highlight the need to shift from pathway-focused manipulation toward integrative regulatory strategies. It should be emphasized that this review does not propose an established regulatory model. Instead, based on currently available evidence, we present a conceptual framework suggesting that selected bHLH TFs may coordinately regulate multiple developmental and metabolic processes at the whole-plant level.

Recent studies demonstrate that specific bHLH TFs coordinate multiple biological processes within a unified regulatory framework. AaMYC3, for example, links GST initiation with artemisinin biosynthesis by activating *AaHD1* and key pathway genes, while interacting with AabHLH1 and AabHLH113 to enhance *ADS* and *DBR2* expression [45]. However, the relatively moderate yield improvement achieved thus far suggests that regulatory thresholds imposed by carbon allocation and metabolic constraints may limit further gains (Figure 4).

AaSPATULA further extends this paradigm from tissue-level coordination to whole-plant integration. By interacting with AaHD8 to activate *AaGSW2*, *AaSPATULA* promotes GST formation while simultaneously enhancing photosynthetic capacity, carbon assimilation, and biomass accumulation [33,61]. Consequently, whole-plant artemisinin yield increases by 2.30–4.16-fold in overexpression lines [82], demonstrating that biomass availability represents a critical limiting factor and providing a mechanistic solution to the “high trichome density–low biomass” trade-off (Figure 4).

Hormone signaling integration constitutes another key layer of this regulatory system. JA and ABA are coordinated through TF networks rather than linear pathways [17,87]. AabHLH113 serves as a convergence node integrating JA and ABA signals via AabHLH112 and AabZIP1, while AaMYC3 further amplifies this network through protein–protein interactions [45,69]. This highlights how bHLH factors enable synergistic crosstalk between hormone pathways to coordinate development and metabolism (Figure 2).

From a broader perspective, similar regulatory strategies are conserved across species. MYC-type TFs such as GoPGF in cotton and SlMYC1 in tomato coordinately regulate glandular structure formation and secondary metabolism, suggesting that developmental–metabolic integration represents a general mechanism in plants [71,72,74,88].

From an applied standpoint, the bHLH regulatory network provides clear advantages for crop improvement. Emerging technologies, including CRISPR/Cas9 and Cut-Dip-Budding systems, enable multi-target editing of regulatory hubs, allowing simultaneous optimization of GST density, metabolic flux, and carbon allocation. Natural variation in regulators such as AaSPATULA further supports the potential of marker-assisted selection and allele-specific engineering for developing high-yielding cultivars (Figure 4).

Despite these advances, key challenges remain. The downstream mechanisms linking bHLH factors to photosynthesis and carbon allocation are still poorly defined, and technical limitations in genetic transformation restrict practical applications in *A. annua*. Future research should focus on constructing system-level regulatory models and integrating multi-omics approaches, including single-cell and spatial transcriptomics, to resolve dynamic regulatory interactions (Figure 4).

Conclusively, the bHLH TF network, exemplified by AaMYC3 and AaSPATULA, represents a shift from pathway-centric engineering toward developmental–metabolic integration. By coordinating trichome development, metabolic flux, and whole-plant physiology, these regulatory hubs provide a foundation for the rational design of high-yielding medicinal plants. Future studies should experimentally validate whether these regulatory relationships operate cooperatively in vivo and determine whether simultaneous manipulation of multiple bHLH TFs can effectively overcome the growth–defense trade-off.

## Data Availability

No new data were created or analyzed in this study. Data sharing is not applicable to this article.
